# An *in vitro* investigation of the hepatic toxicity of PEGylated polymeric redox responsive nanoparticles

**DOI:** 10.1039/d2ra00395c

**Published:** 2022-04-27

**Authors:** Leagh G. Powell, Cameron Alexander, Vicki Stone, Helinor J. Johnston, Claudia Conte

**Affiliations:** Nano Safety Research Group, School of Engineering and Physical Sciences, Heriot-Watt University UK; Division of Molecular Therapeutics and Formulation, School of Pharmacy, University of Nottingham UK; Department of Pharmacy, University of Naples Naples Italy claudia.conte@unina.it

## Abstract

It can be challenging to deliver drugs to cancer cells in a targeted manner at an effective dose. Polymeric nanoparticles (NPs) are promising drug delivery systems that can be targeted to cancer cells using redox responsive elements. More specifically, intracellular and extracellular levels of the antioxidant glutathione (GSH) are elevated in cancer cells and therefore the use of NPs with a cleavable GSH-responsive element allowing these NPs to target cancer cells and trigger the release of their cargo (*e.g.* anticancer drugs). The aim of this study was to assess the hepatotoxicity of polymeric NP delivery systems with and without a redox sensitive element. Copolymer poly (lactic-*co*-glycolic acid) (PLGA) and polyethylene glycol (PEG) NPs with (RR-NPs) and without (nRR-NPs) a redox responsive dithiylethanoate ester linker were synthesised and their toxicity assessed *in vitro*. As the liver is a primary site of NP accumulation, the C3A hepatocyte cell line was used to assess NP toxicity *in vitro via* investigation of cytotoxicity, cytokine production, genotoxicity, intracellular reactive oxygen species (ROS) production, intracellular calcium concentration, and hepatocyte function (albumin and urea production). The cellular uptake of NPs was also assessed as this may influence the cellular dose and, therefore, the cellular response. Both NPs had no detrimental impact on cell viability. However, both NPs stimulated an increase in cytokine (IL-1ra) and ROS production and decreased hepatocyte function, with the greatest effect observed for nRR-NPs. Only nRR-NPs caused DNA damage. Cells internalised both NPs and caused a (sub-lethal) increase in intracellular calcium levels. Therefore, whilst the NPs did not have a negative impact on cell viability, the NPs were able to elicit sub-lethal toxicity. By using a battery of tests we were able to demonstrate that RR-NPs may be less toxic than nRR-NPs. Our findings can therefore feed into the development of safer and more effective nanomedicines and into the design of testing strategies to assess polymeric NP safety based on knowledge of their mechanism of toxicity.

## Abstract 2

PEGylated polymeric RR-NPs are less toxic than nRR-NPs. Both formulations did not have a negative impact on cell viability, but were able to elicit sub-lethal toxicity, especially in the case of non redox responsive NPs.

## Graphical abstract

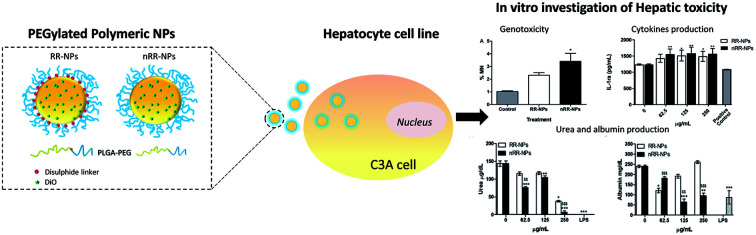

## Introduction

The improved targeting of drugs to cancer cells is essential to enhance delivery and minimise adverse effects. Polymeric nanoparticles (NPs) can be used as delivery vehicles to enable the transport and protection of anti-cancer drugs.^1,2^ Elements can be introduced into the design of such NPs to improve targeting to cancer cells. For example, the incorporation of redox responsive elements into polymeric NPs can improve targeting to cancer cells and trigger the release of cargo due to elevated levels of the antioxidant glutathione (GSH) in tumours.^3,4^ Coatings can also be used, such as polyethylene glycol (PEG), to give to polymeric NPs “stealth” like properties, protecting sensitive cargo and increasing circulation time in the body.^5^

The polymer Poly-lactic-*co*-glycolic acid (PLGA) has been used to generate NPs for drug delivery due to its biodegradability and previous use in Food and Drug Administration (FDA) approved medical devices.^6^ However, when PLGA NPs undergo hydrolytic degradation, the by-products, poly-lactic acid and polyglycolic acid, have been shown to cause cytotoxicity and stimulate elevated levels of cytokine and reactive oxygen species (ROS) production by cells (*e.g.*, macrophages) *in vitro*.^7,8^ Therefore, it is essential to perform a comprehensive assessment of the toxicity of PLGA NPs in parallel to investigating efficacy.

We generated PLGA-PEG NPs with (RR-NPs) and without (nRR-NPs) a novel redox-responsive disulphide linker at an ethanoate ester between the PLGA core and the PEG shell. Cleavage of the disulphide linker is expected in environments with elevated levels of GSH (*e.g.* tumours) to promote targeting to cancer cells and to release the cargo. We have previously demonstrated that the inclusion of this redox sensitive element leads to better delivery of anti-cancer drugs in both 2-D and 3-D lung cancer cellular models.^4^

From a translational point of view the vast numbers, diversity, and complexity of polymeric NPs in development means testing each NP *in vivo* is impractical and prohibitively high in the investment of resources. Furthermore, due to the ethical implications of performing animal research and the inability of animal models to predict human responses accurately, there is a drive to reduce the reliance placed on rodent testing in toxicology.^9,10^ Therefore, *in vitro* testing allows for a more ethical, relatively rapid and cost-effective assessment of NP toxicity. Furthermore, cells of human origin can be used to overcome issues with species differences.

It is well recognised that NPs accumulate within the liver independently of the administration route.^11,12^ Accordingly, this study aimed to investigate the toxicity of RR-NPs and nRR-NPs to the liver using the human C3A hepatocyte cell line. This cell line was selected as it has been previously demonstrated to provide similar responses to primary rat and human hepatocytes.^13–15^

When assessing the toxicity of polymeric NPs *in vitro*, testing can often be limited to investigation of cytotoxicity. However, a more robust assessment of the toxicity of polymeric NPs is required that encompasses assessment of lethal and sub-lethal responses. Improved knowledge of the mechanism of polymeric NP toxicity can feed into the design of testing strategies which screen their toxicity.

The study aimed to compare the hepatic toxicity of RR-NPs and nRR-NPs using the human C3A hepatocyte cell line to identify if they could potentially be used as new delivery vehicles. A broad range of endpoints were investigated to assess their safety and probe their mechanism of toxicity. First, the impact of NPs on cell viability was evaluated, which allowed the toxic potency of NPs to be assessed and sub-lethal concentrations of NPs to be identified. Next, sub-lethal markers of toxicity were assessed including cytokine production (Interleukin (IL)-8, IL-6, IL-1β, and IL-1 receptor antagonist (IL-1ra)), intracellular levels of calcium and reactive oxygen species (ROS), genotoxicity and liver function (urea and albumin production). Finally, NP internalisation was investigated as their interaction with cells may influence the cellular dose and therefore the response that is activated.

## Experimental

### Materials

All materials and reagents were of analytical or HPLC grade and were purchased from Sigma-Aldrich (Dorset, UK) unless stated otherwise.

### Nanoparticle preparation

A non-redox responsive PLGA-PEG block copolymer and a redox reactive PLGA-ss-PEG block copolymer were synthesised and used to generate core–shell NPs as previously described^4^ and loaded with green, fluorescent 3,3′-dioctadecyloxacarbocyanine perchlorate (DiO) (Life Technologies, UK) at 0.1% w/w respect to copolymer weight. NPs were prepared at a concentration of 1 mg mL^−1^ in sterile water, filter sterilised and stored at 4 °C for a maximum of 4 weeks. For all experiments, NPs were dispersed in relevant media at appropriate concentrations, briefly vortexed and used immediately.

### Nanoparticle characterisation

NPs were characterised by dynamic light scattering (DLS) and transmission electron microscopy (TEM). We measured hydrodynamic diameter, polydispersity index (PDI) and zeta potential (*ζ*) of freshly prepared NPs using a Zetasizer Nano ZS (Malvern, Malvern, UK) at a NP concentration of 125 μg mL^−1^. Three different batches of NPs were assessed on 3 separate occasions. The morphology of the NPs was examined by transmission electron microscopy (TEM). Samples were imaged using a JEOL (JEM-10-10) electron microscope. A few drops were added onto a copper grid and allowed to dry in air. NPs were negatively stained with 2% phosphotungstic acid solution.

To assess the properties of the NPs in cell culture media, NPs were dispersed in a phenol-red free (PRF) Minimum Essential Medium Eagle (MEM) cell culture medium containing 10% heat-inactivated foetal bovine serum (FBS), 2 mM l-glutamine (Gibco, ThermoFisher, Loughborough UK), 100 U mL^−1^ penicillin/streptomycin (Gibco), 1 mM sodium pyruvate and 1% non-essential amino acids (termed phenol red-free (PRF)-Complete Medium) at a concentration of 125 μg mL^−1^. Hydrodynamic diameter, PDI and *ζ* measurements were performed at the time of preparation (T0) and 24 h post-incubation at 37 °C and 5% CO_2_ (T24). Three different batches of NPs were assessed on 3 separate occasions.

Encapsulation studies were performed using DiO loaded NPs, solubilised in dichloromethane (DCM) (1 mg mL^−1^) and analysed for DiO content by UV spectrophotometry at 488 nm. The concentration of DiO was calculated utilising a standard calibration curve derived for DCM solutions of DiO (0.5–60 μg mL^−1^). To verify a possible interference of copolymers on DiO quantitative analysis, unloaded NPs were dissolved in DCM and analysed under the same conditions reported for DiO.

### Cell culture

Human C3A hepatocellular carcinoma cells (American Type Culture Collection) were cultured in MEM medium supplemented with 10% heat-inactivated foetal calf serum (FCS), 2 mM l-glutamine, 100 U mL^−1^ penicillin/streptomycin, 1 mM sodium pyruvate and 1% non-essential amino acids (termed Complete Medium) and incubated at 37 °C and 5% CO_2_.

### Cytotoxicity

The viability of C3A cells following exposure to NPs was assessed simultaneously using the Alamar blue (AB) assay (for metabolic activity) and the Neutral red (NR) assay (for lysosomal function) adapted from the method described by Connolly *et al.*^16^ Briefly, cells were seeded at 1.56 × 10^5^ cells per cm^2^ in 96-well plates and incubated for 24 h at 37 °C and 5% CO_2_. Cells were then exposed to NPs (5–250 μg mL^−1^), Complete Medium (negative control), 1% Triton X-100 (positive control) or sterile water (vehicle control) diluted in Complete Medium for 24 h at 37 °C and 5% CO_2_. Following NP treatment, the cell supernatant was collected and frozen at −80 °C for further cytokine analysis, and cells were washed twice using Phosphate-Buffered Saline (PBS). The AB reagent (1.25% v/v AB, ThermoFisher) was prepared in PRF Complete Medium, added to cells, and incubated at 37 °C and 5% CO_2_ for 1 h. Following this, fluorescence was measured on a SpectraMax M5 Microplate Reader (Molecular Devices, Berkshire, UK) at excitation/emission (Ex/Em) of 532/590 nm. Cells were then washed twice using PBS, and the NR solution (33 μg mL^−1^, in PRF Complete Medium) was added to each well and incubated at 37 °C and 5% CO_2_ for 1 h. Following incubation, cells were washed with PBS three times, and a solution of 50% ethanol and 1% acetic acid prepared in deionised water was added before shaking for 20 minutes at room temperature (RT). Fluorescence was measured at Ex/Em of 532/645 nm. Data are expressed as mean % cell viability (*i.e.*, % of negative control).

### Cell internalization

A 96-well plate-based assay was developed to determine the uptake of NPs by C3A cells. Cells were seeded at 1.56 × 10^5^ cells per cm^2^ in 96-well plates and incubated for 24 h at 37 °C and 5% CO_2_. Cells were then exposed to NPs at concentrations ranging from 5–250 μg mL^−1^ or Complete Medium (negative control) in duplicate for 10 minutes, 60 minutes or 1440 minutes (24 h) at 37 °C and 5% CO_2_. Following NP exposure, cells were washed with PBS and incubated at 37 °C and 5% CO_2_ for 10 minutes in 50 μL of 0.4% Trypan Blue to quench extracellular fluorescence. Following this, cells were washed four times with PBS and lysed using a solution of 0.2% Triton X-100 in sterile water. The concentration of NPs (μg mL^−1^) retained after NP treatment was calculated utilising a standard calibration curve derived for NPs (0.3–50 μg mL^−1^) prepared in the cell lysate of untreated cells and measured at Ex/Em 488/526 nm. Data are expressed as mean NP (μg mL^−1^) retained in the cells.

Confocal microscopy was used to visualise NP uptake by C3A cells. A single time point (1440 minutes (24 h)) was selected based on the findings from the above plate-based method. Cells were seeded onto 12 mm uncoated glass coverslips in the wells of a 24-well plate at 6.58 × 10^4^ cells per cm^2^ and incubated at 37 °C and 5% CO_2_ for 24 h. Cells were then exposed to 125 μg mL^−1^ of NPs or complete medium (control) for 24 h at 37 °C and 5% CO_2_. Following this, cells were washed with PBS and fixed in 3% formaldehyde for 30 minutes at 4 °C. Subsequently, the cells were washed with PBS and incubated with 50 mM ammonium chloride to quench unreacted aldehyde groups for 10 minutes at RT. Cells were then washed with PBS and permeabilized with 0.1% Triton for 20 minutes at RT. Cells were washed again with PBS and incubated with the primary antibody, monoclonal anti α tubulin mouse ascites fluid clone DM1A (ThermoFisher) (1 : 200 in PBS), for 1 h at RT. After incubation, cells were washed and incubated with the secondary antibody, Rhodamine Red goat anti-mouse Immunoglobulin G (ThermoFisher) (1 : 100 in PBS), for 1 h at RT. Finally, cells were washed, and coverslips were mounted onto glass slides with Vector shield mounting media (Vector Laboratories, Peterborough, UK) and edges sealed with clear varnish. Cells were imaged using an SP5 SMD gated-STED confocal laser scanning microscope (Leica, Milton Keynes, UK) and the Leica Application Suite program (Leica).

### Measurement of cytokine production

A multiplex sandwich ELISA was used to measure the secretion of IL-8, IL-1ra, IL-1β and IL-6 from C3A cells exposed to NPs. Supernatants obtained from the cytotoxicity experiments were thawed and analysed using a human magnetic Luminex assay kit (R&D Systems, Abingdon, UK), as per the manufacturer's instructions. All steps were carried out at RT and in subdued lighting conditions. Sublethal NP concentrations (31.25, 62.5 and 125 μg mL^−1^) were assessed, as well as Complete Medium (negative control) or 10 ng mL^−1^ of recombinant human TNF-α (rhTNF-α). Briefly, the microbead cocktail was added to appropriate wells in a 96-well plate, followed by samples, negative controls and standards (0–2000 pg mL^−1^ prepared in Complete Medium) and incubated for two hrs shaking at RT. After incubation, wells were washed three times with wash buffer, before adding biotin antibody cocktail and incubation for one hr while shaking at RT. Wells were washed three times with wash buffer, followed by the addition of streptavidin-PE, and incubated 30 minutes at RT with constant shaking. Before measuring, the wells were washed three times with wash buffer, and finally, samples were resuspended in wash buffer and read using Bio-Plex MAGPIX Multiplex Reader (Bio-Rad). The concentration of cytokines present in the samples was calculated from the linear regression obtained from the standard curves. Data are expressed as average pg mL^−1^ ± SEM.

### Genotoxicity assessment

Oxidative DNA damage and DNA strand breaks were assessed using the formamidopyrimidine DNA glycosylase (Fpg) modified Comet assay with a 24-well Comet chip, according to manufacturer's guidelines (Trevigen, Abingdon, UK). Analysis of the fragments was assessed using the repair enzymes (FLARE) assay kit (Trevigen). Cells were seeded at 1.56 × 10^5^ cells per cm^2^ and incubated for 24 h at 37 °C and 5% CO_2_.

The cells were then washed with Hank's balanced salt solution (HBSS) and exposed to NPs (125 and 250 μg mL^−1^), Complete Medium (negative control) or 60 μM hydrogen peroxide (H_2_O_2_, positive control) in duplicate, for 1440 minutes at 37 °C and 5% CO_2_. Cells were then washed, trypsinized and resuspended in Complete Medium. Following this, cells were centrifuged, the supernatant discarded, and the pellet resuspended in ice-cold PBS. Cells were then added to low melting point agarose, deposited onto a 24-well Comet chip and incubated on ice for 15 minutes before being transferred to a lysis solution for 12 h at 4 °C. Then slides were immersed in the FLARE buffer for 30 minutes and incubated with Fpg enzyme (1 : 75 dilution in the Fpg reaction buffer) or Fpg reaction buffer alone at 37 °C for 30 minutes. Slides were then allowed to equilibrate for 20 minutes in electrophoresis solution at 4 °C. Electrophoresis was performed for 40 minutes at 30 V and 300 mA at 4 °C. Next, slides were washed with deionised water and then with 70% ethanol. Samples were stained with GelRed (Biotum, Fremont, USA) to visualise DNA and imaged using a fluorescence microscope (AX10 Microscope (Zeiss, Cambridge, UK) with a Stingray camera, Allied Vision Technologies, Stadtroda, Germany) connected to image-analysing software (Comet Assay IV, Perceptive Instruments, St Edmunds, UK). Samples were coded and scored blind. Fifty measurements were taken for each slide. Data are expressed as mean % Tail DNA ± SEM.

A 24-well based Micronucleus assay was used to assess DNA damage to verify results from the Comet assay. Following 24 h growth at 37 °C and 5% CO_2_ in 24-well plates (1.56 × 10^5^ cells per mL), C3A cells were washed twice with PBS then exposed to NPs (125 μg mL^−1^), or Complete Medium (control), in duplicate for 24 h at 37 °C and 5% CO_2_. Cells were washed twice with PBS and exposed to a cytokine blocker Cyto-B (6 μg mL^−1^) prepared in Complete Medium for a further 36 h to allow for the identification of cells that have divided post-NP exposure. Subsequently, the supernatant was discarded, cells were washed twice with PBS and trypsinized. Following this, cells were centrifuged at 2000 g for 1 minute and washed an additional 2 times with PBS and finally resuspended in ice-cold PBS. Cells were then added to a Cytospin (Shandon) and spun at 1500 g for 5 minutes. Slides were air-dried and fixed for 10 minutes in ice-cold 90% methanol. Cells were stained with 20% Giemsa stain (VWR) for 6 minutes then washed twice with PBS. Following air drying for 4–5 h, slides were dipped in Xylene for 10 seconds and allowed to dry.

Micronuclei were counted only in binucleated cells confirmed by light microscopy (Zeiss AX10 with Allied Vision Technologies Stingray camera). A total of 500–1000 cells were analysed. For each treatment, data is represented as % micronucleus ((micronucleus observed ÷ total number of cells) × 100) mean.

### Measurement of cellular ROS production

A 96 well-plate fluorescence-based assay was used to determine ROS production by C3A cells following NP exposure. Cells were seeded 1.56 × 10^5^ cells per cm^2^ in 96-well plates and incubated for 24 h at 37 °C and 5% CO_2_. Following this, cells were pre-treated with Trolox (100 μM) (6-hydroxy-2,5,6,7,8-tetramethylchroman-2-carboxylic acid) in Complete Medium or Complete Medium alone, for 1 h at 37 °C and 5% CO_2_. Following pre-treatment, cells were washed twice using PBS. Cells were then exposed to NPs (62.5, 125 and 250 μg mL^−1^), Ultrafine Carbon Black (UFCB-Printex-90, 10 μg mL^−1^, positive control) or Complete Medium (negative control) for 24 h at 37 °C and 5% CO_2_.

Following treatment, cells were washed twice using HBSS, then incubated for 5 h in methanol containing 5 μM 2′,7′-dichlorofluorescein-diacetate (DCFH-DA) or in methanol without DCFH-DA in reduced light at RT, enabling the detection of possible NP interference. Fluorescence was measured at Ex/Em 495/529 nm. Data expressed as a mean fold increase compared to Complete Medium (negative control).

### Measurement of intracellular calcium

The fluorescent dye Fura-2 was used to determine intracellular calcium concentration in C3A cells. When Fura-2-AM dye penetrates cells, esterases cleave the dye, trapping it in the cell where the maximal excitation is differential for unbound dye (380 nm) and calcium bound dye (340 nm).^17^ Cells were seeded at 1.56 × 10^5^ cells per cm^2^ in a 96-well black plate with an optic bottom and incubated for 24 h at 37 °C and 5% CO_2_. Following this, cells were washed twice with PBS. Cells were pre-treated with serum-free MEM medium containing Probenecid (1 mM), Pluronic F127 (16 mM), HEPES (10 mM) with or without Fura-2-AM (1 μM) for 1 h at 37 °C and 5% CO_2_. Cells were washed once with a serum-free MEM medium containing HEPES (10 mM).

Before NP exposure, background fluorescence at Ex/Em 340/510 nm and 380/510 nm were measured (T0). Cells were then exposed to NPs at 125 μg mL^−1^, Ultrafine Carbon Black (UFCB-Printex-90, 10 μg mL^−1^ (positive control)), or Complete Medium (negative control). Fluorescence was measured every 2 minutes for 36 minutes. Data are represented as the mean ratio of fluorescence at Ex/Em 340/510 nm and 380/510 nm.

### Urea and albumin production

The QuantiChrom Urea assay kit (ThermoFisher) was used to assess urea production as per the manufacturer's guidelines. Cells were seeded at 1.56 × 10^5^ cells per cm^2^ in 96-well plates and incubated for 24 h at 37 °C and 5% CO_2_. Following this, cells were exposed to NPs (62.5, 125 and 250 μg mL^−1^) or Complete Medium (negative control) for 24 h at 37 °C and 5% CO_2_. Data are expressed as average μg dL^−1^ of urea, calculated from a urea standard curve (3.5–500 μg dL^−1^).

Bromocresol green was used to quantify albumin production in C3A cells. Cells were seeded at 1.56 × 10^5^ cells per cm^2^ in 96-well plates and incubated for 24 h at 37 °C and 5% CO_2_. Then, cells were exposed to NPs (62.5, 125 and 250 μg mL^−1^), E. coli-derived lipopolysaccharide (LPS) 100 ng mL^−1^ or Complete Medium (negative control) for 24 h at 37 °C and 5% CO_2_. Cell supernatants were collected and incubated with Bromocresol green solution (0.066 mM in 100 mM Succinate Buffer, pH 4.2) for 5 minutes shaking at RT. Absorbance was measured at 630 nm. Data were expressed as average albumin concentration (mg dL^−1^), calculated from an albumin standard curve (6.25–400 mg dL^−1^).

### Statistical analysis

Experimental data were analyzed using GraphPad Prism software. One-way ANOVA using Tukey post-test or two-way ANOVA followed by the Bonferroni post-test were used to test for statistical significance. Significance was set at *p* < 0.05. All experiments were performed in triplicate and repeated a minimum of 3 times on separate occasions unless otherwise stated.

## Results and discussion

### Characterisation of NPs

**Fig. 1:**
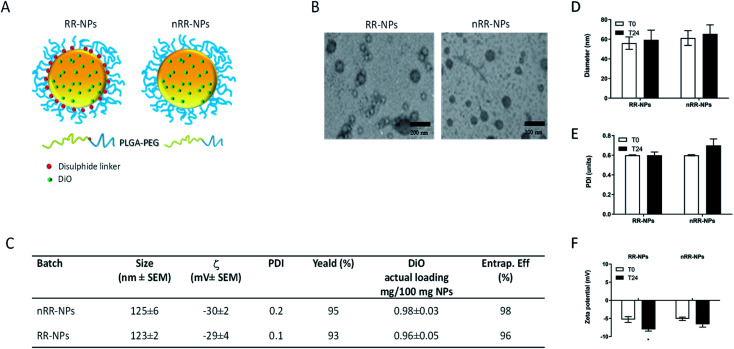
Physicochemical characterization of RR-NPs and nRR-NPs: schematic illustration of RR-NPs and nRR-NPs (A). TEM images of freshly prepared NPs (scale bar is 200 nm) (B). Table summarizing the colloidal properties of NPs loaded with DiO freshly dispersed in MilliQ water (C). Hydrodynamic diameter (D), polydispersity index (PDI) (E) and *ζ* (F) of NPs dispersed in Complete Medium (125 μg mL^−1^) at 0 h (T0) and 24 h (T24) post-incubation at 37 °C, 5% CO_2_. Data are expressed as mean ± SEM (*n* = 3).

In this paper, we prepared PEGylated redox responsive (RR-NPs) and non-redox responsive NPs (nRR-NPs) with a core–shell structure, loaded with DiO as a fluorescent and lipophilic model drug (Fig. 1A). Properties of freshly prepared NPs dispersed in MilliQ water are reported in the table in Fig. 1C. NPs showed a hydrodynamic diameter of ∼100 nm, a low polydispersity index, and negative *ζ* values. A high yield of NP production demonstrated that no copolymer/drug precipitation occurred during their fabrication. DiO loading was 1 mg/100 mg NPs, corresponding to a complete entrapment efficiency. A spherical morphology is demonstrated by TEM images (Fig. 1B), despite TEM images of the nRR-NPs appear monodispersed, whereas RR-NPs appeared to have two different size populations. Therefore, it is possible that RR-NPs may have lost some of the PEG coating from the PLGA core resulting in a reduction in size. The redox responsiveness of the RR-NPs compared to nRR-NPs was previously demonstrated^4^ by incubating the carriers in GSH 5 mM. In fact, in conditions simulating intracellular reductive environments, we observed a fast disassembly of the external PEG shell of the RR-NPs, thus triggering on-demand drug release. The physicochemical properties of the RR-NPs and nRR-NPs were also characterized in cell culture medium to reflect conditions relevant to the toxicity assessment. The hydrodynamic diameter of RR-NPs and nRR-NPs was assessed immediately after preparation in Complete Medium (T0) and 24 h post-incubation at 37 °C and 5% CO_2_ (T24). When compared NPs prepared in MilliQ water the hydrodynamic diameter of both NPs was less in cell culture medium, while increased for PDI, and decreased for *ζ* values (Fig. 1D–F), thus suggesting a slight agglomeration of the NPs in cell culture medium, probably due to the presence of proteins. However, the properties of the NPs in cell culture medium remained relatively unchanged at both time points (T0 and T24), thus suggesting that both NP dispersions are relatively stable over time.

### Cytotoxicity

**Fig. 2:**
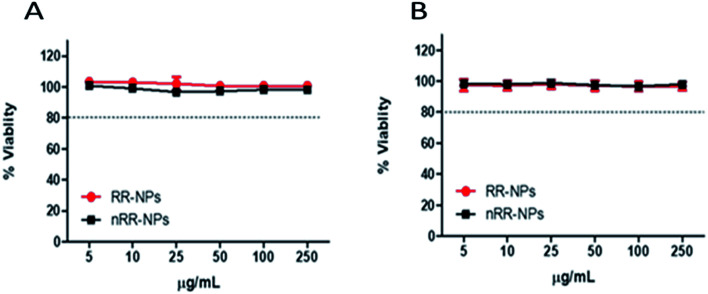
Toxicity of RR-NPs and nRR-NPs to C3A hepatocytes *in vitro*. Cytotoxicity was assessed *via* the AB (A) and NR (B) assays following cell exposure to RR-NPs or nRR-NPs at concentrations of 5–250 μg mL^−1^ for 24 h. Data are expressed as mean % viability (*i.e.*, % of untreated control) ± SEM (*n* = 3).

Both cytotoxicity assays gave similar results for both NPs, showing relatively low cytotoxicity with less than a 10% reduction in cell viability observed at all concentrations tested, 24 h post-exposure (Fig. 2A and B). An EC_20_, the concentration where NP exposure leads to 20% cell death, could not be calculated for these NPs for either assay, suggesting that these NPs had relatively low cytotoxicity. Interestingly, these NPs also induced low levels of cytotoxicity in other cell types, such as lung cancer cell lines (A549 and Calu-3).^4,18^ Similar to our findings, Aranda *et al.*^19^ demonstrated that PLGA- Poly(ethylene oxide) (PEO) NPs with a similar composition to the RR-NPs and nRR-NPs used in our study, exhibited similarly low levels of cytotoxicity to primary rat hepatocytes.

### Cell internalization

**Fig. 3:**
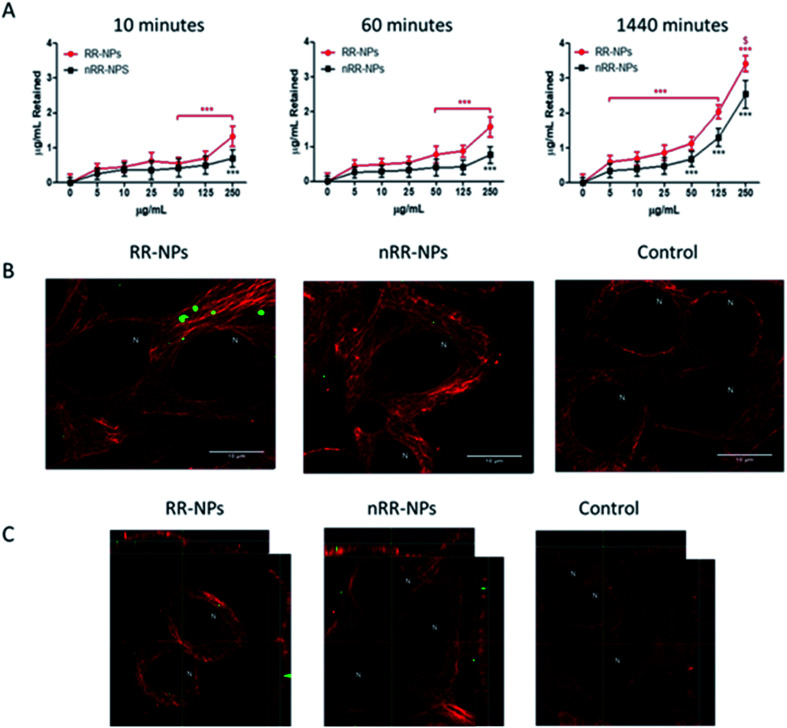
Uptake of RR-NPs and nRR-NPs by C3A hepatocytes over time. For the plate-based method, C3A cells were exposed to RR-NPs or nRR-NPs for 10, 60 or 1440 minutes, at 5–250 μg mL^−1^ or Complete Medium (0 μg mL^−1^ negative control) (A). Data are expressed as mean μg mL^−1^ retained in cells after quenching (*n* = 3). Significance indicated by *** = *p* < 0.001 compared with control. Significance indicated by $ = *p* < 0.05 for RR-NPs compared with nRR-NPs at the same concentration and time point. For confocal microscopy, C3A cells were exposed to RR-NPs and nRR-NPs (green) at 125 μg mL^−1^ or Complete Medium (negative control) for 1440 minutes (B). Z-stacks xy and yz micrographs were generated to confirm uptake into the cell interior (C). Representative images are presented (*n* = 3). Tubulin (red) and N indicates the location of the nucleus, scale bar = 10 μm.

There was a significant concentration and time-dependent increase in the internalization of both RR-NPs and nRR-NPs by hepatocytes (Fig. 3A). The level of uptake of RR-NPs was significantly higher than that observed for nRR-NPs, which is in line with the extracellular responsiveness demonstrated previously for A549 cells, where there was higher uptake of these redox responsive NPs.^4^ The highest level of uptake by cells was observed for both NPs at a concentration of 125 μg mL^−1^ at 1440 minutes (24 h). Therefore, confocal microscopy was performed to visualize uptake under these conditions. Although both NPs were internalised by C3A cells (Fig. 3B) and confirmed in the xy-yz micrographs generated from z-stacks (Fig. 3C), overall cellular uptake appears to be relatively low. Interestingly, similar levels of uptake of PEGylated polyester (polycaprolactone-poly (dimethylamino ethyl methacrylate)) NPs were observed in hepatocyte HepG2 cells.^20^ The NPs within the C3A cells appear to be compartmentalised, potentially between cells in bile canaliculi or within organelles such as endosomes, lysosomes or mitochondria. There is an absence of NP localisation within the nucleus of C3A cells. Further work will be required to confirm the intracellular fate of the NPs. Notably, previous studies have demonstrated that polystyrene NPs localised within the mitochondria and bile canaliculi of hepatocytes.^15^

### Cytokine production

Assessment of cytokine production is commonly used to investigate the inflammatory response stimulated by NPs *in vitro*.^21^

The NPs investigated did not stimulate pro-inflammatory cytokine production. The data for IL-8, IL-1β and IL-6 is therefore not presented as the level of production was below the limit of detection for all treatment groups.

Our findings align with other studies investigating cytokine production by cells following exposure to polymeric NPs. For example, no increase in IL-1β, IL-6, TNF-α or IL-8 cytokine production by monocytes and polymorphonuclear cells was observed following exposure to PLGA-PEG NPs *in vitro*.^22^ In contrast to our observations, some *in vitro* studies show that polymeric NPs can stimulate cytokine production such as PLGA NPs shown to activate short term pulmonary inflammatory responses (*e.g.*, neutrophil accumulation, TNFα and MCP-1 production) in rats following intratracheal instillation, in particle size and charge related manner.^23^ However, a large dose of NPs (5 mg kg^−1^) was administered in this study which may explain why an inflammatory response was observed. Increased serum levels of TNF-α and IL-6 were observed after oral exposure to polymeric NPs as polyurethane NPs and *N*-isopropylacrylamide-vinylpyrrolidone-acrylic acid NPs.^24^ The discrepancies between the findings of different studies is likely to be due to differences in their experimental design (*e.g.* model used, physico-chemical properties of the NPs, concentrations of NPs tested, time points assessed).

**Fig. 4:**
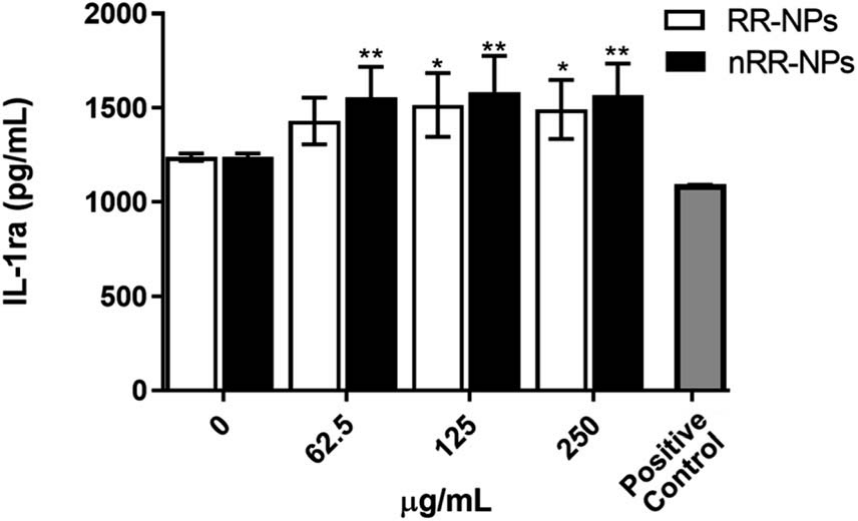
Cytokine production (IL-1ra) by C3A cells following exposure to RR-NPs and nRR-NPs. Cytokine (IL-1ra) production was measured following cell exposure to RR-NPs and nRR-NPs (62.5, 125 and 250 μg mL^−1^), Complete Medium (0 μg mL^−1^, negative control) or hrTNF-α 10 ng mL^−1^ (positive control) for 24 h. Data are expressed as mean cytokine production (pg mL^−1^) ± SEM (*n* = 3). Significance indicated by * = *p* < 0.05 and ** = *p* < 0.005 when compared to negative control.

Interestingly, we observed a significant increase in the production of the anti-inflammatory cytokine IL-1ra (Fig. 4) at all concentrations for nRR-NPs and concentrations of 125 and 250 μg mL^−1^ for RR-NPs. Although not often investigated in nanotoxicology studies, IL-1ra is an inhibitor of the proinflammatory activity of IL-1 cytokines and is therefore involved in resolving inflammatory responses. As experimental studies typically focus on investigating the production of proinflammatory cytokines, we suggest that the production of IL-1ra is assessed more widely when screening NP toxicity to enhance the understanding of the dynamics of inflammatory responses that NPs activate.

### Genotoxicity

**Fig. 5:**
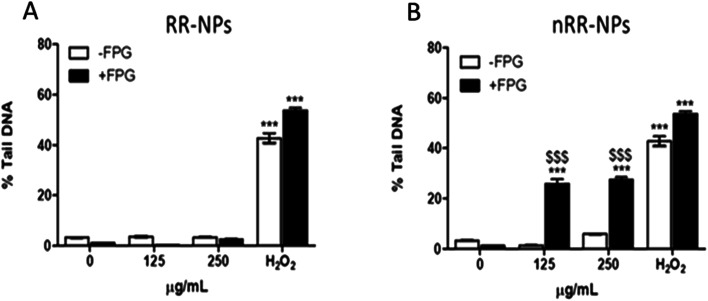
Genotoxicity in C3A cells following exposure to RR-NPs and nRR-NPs. For assessment of genotoxicity, C3A cells were exposed to RR-NPs (A) and nRR-NPs (B) at 125 and 250 μg mL^−1^, 60 μM H_2_O_2_ (positive control) or Complete Medium (0 μg mL^−1^ negative control) for 24 h and genotoxicity was assessed using the Comet assay in the presence (+) and absence (−) of Fpg. Data are expressed as mean % Tail DNA ± SEM (*n* = 3). Significance indicated by *** = *p* < 0.001 compared with the negative control. Significance indicated by $$$ = *p* < 0.001 for nRR-NPs compared to RR-NPs at the same concentration with Fpg.

The Comet assay assessed whether RR-NPs and nRR-NPs (125 μg mL^−1^ and 250 μg mL^−1^ for 24 h) induced DNA damage in C3A cells. The potential contribution of oxidative stress to DNA damage was assessed by conducting the assay with and without the addition of Fpg. This repair enzyme recognizes and cleaves oxidized bases in damaged DNA.^25^ RR-NPs did not induce DNA damage (Fig. 5A). The nRR-NPs induced a significant increase in DNA damage in the presence of Fpg at both concentrations, suggesting DNA damage may be caused by an oxidative mechanism (Fig. 5B). Future studies could assess a wider concentration range of NPs to identify the threshold for toxicity in this assay. The positive control H_2_O_2_ stimulated a significant increase in DNA damage in the presence and absence of Fpg (Fig. 5).

**Fig. 6:**
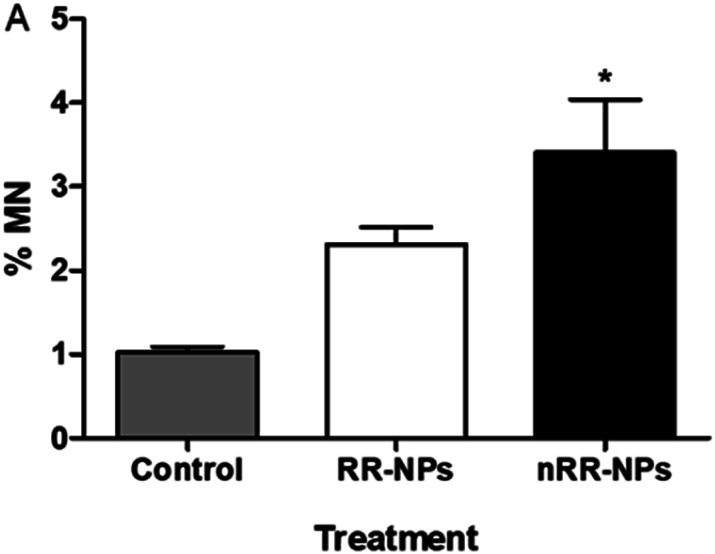
Genotoxicity of RR and nRR-NPs in C3A cells using Micronucleus assay. C3A cells were exposed to RR-NPs and nRR-NPs at 125 μg mL^−1^ and Complete Medium (0 μg mL^−1^ negative control) for 24 h. Data expressed as average % micronucleus (MN) ± SEM (*n* = 3). Significance * = *p* < 0.05 when compared with negative control.

Chromosomes that are fragmented or unable to migrate with the other chromosomes in the cell can generate micronuclei, an indicator of DNA damage.^25^ The micronucleus assay was therefore also used to assess the genotoxicity of RR-NPs and nRR-NPs following exposure of C3A cells at a concentration of 125 μg mL^−1^ for 24 h. As seen in the Comet Assay, RR-NPs do not induce DNA damage (Fig. 6). However, the nRR-NPs induced a significant increase in the % of micronuclei observed. Prior studies using the sister chromatid exchange assay indicated that PLGA-PEG NPs with a similar composition to the nRR-NPs, induced genotoxicity in Chinese hamster ovary cells *in vitro*.^26^ Additionally, *in vitro* studies using the Comet assay showed that PLGA-PEO NPs were genotoxic to primary rat hepatocytes and Kupffer cells.^27^ Moreover, mononucleated TK6 (human B-lymphoblastoid) cells exposed to PLGA-PEO NPs have stimulated micronuclei formation, which indicates that they cause DNA damage.^28^ In contrast, some studies show a lack of genotoxicity by PLGA NPs. For example, Setyawati *et al.* demonstrated that NP-mediated genotoxicity *in vitro* was dependent on cell type and NP surface coating, such as PEG.^29^

The lack of agreement between different studies is likely to be due to the use of NPs with different physicochemical properties, and the use of different cell types, NP concentrations, time points and selection of different assays to assess genotoxicity.

### Cellular ROS production

**Fig. 7:**
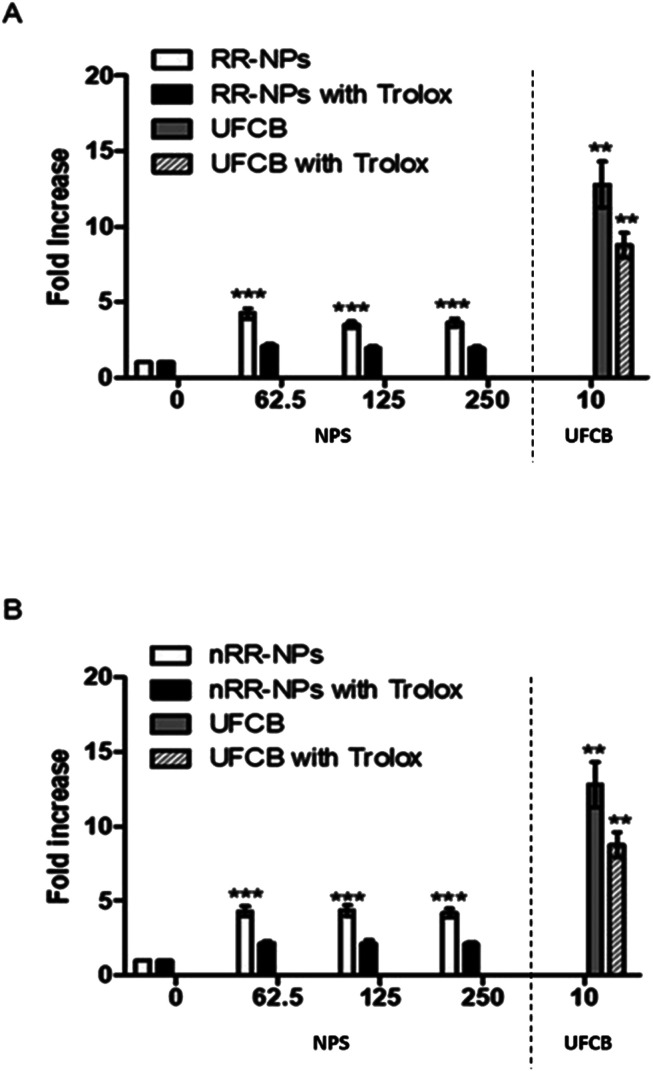
Measurement of intracellular ROS production. C3A cells were exposed to RR-NPs (A) and nRR-NPs (B) at 62.5, 125 and 250 μg mL^−1^, UFCB 10 μg mL^−1^ (positive control) or Complete Medium (0 μg mL^−1^, negative control) for 24 h in the presence and absence of Trolox. Values represent mean fold increase (in fluorescence) ± SEM (minimum of *n* = 3), significance indicated by *** = *p* < 0.001 treatments compared with the untreated control.

Stimulation of oxidative stress has been identified as an important mechanism by which NPs cause toxicity.^30^ It was investigated whether RR-NPs and nRR-NPs stimulated intracellular ROS production by C3A cells using the DCFH-DA assay. A significant increase in ROS production in C3A cells was observed for RR-NPs and nRR-NPs at all concentrations tested (Fig. 7A and B). Additionally, for both NPs, cells not pre-treated with the antioxidant Trolox had significantly higher levels of ROS production than Trolox pre-treated cells. This indicates that Trolox could provide protection against NP mediated ROS production. As there appears to be no concentration dependent element to this response it would be important to investigate ROS production at lower concentrations in the future. Furthermore, confirmation of whether oxidative stress was activated in cells following NP exposure could be investigated using a wider range of endpoints (*e.g.*, depletion of antioxidants, assessment of markers of oxidative damage).

### Intracellular calcium

**Fig. 8:**
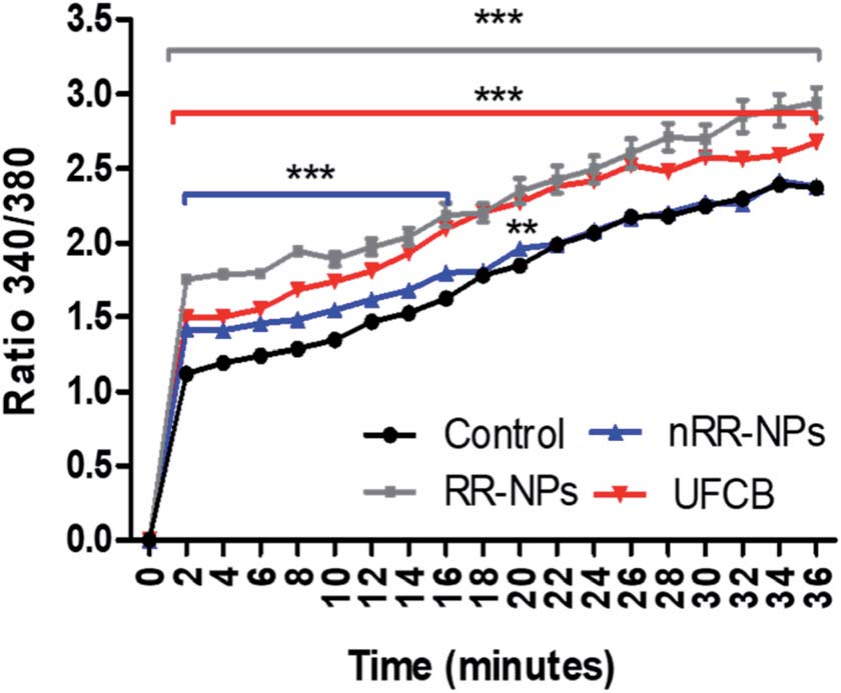
Measurement of intracellular Calcium in C3A cells exposed to nRR-NPs and RR-NPs. The cells were exposed to UFCB 5 μg mL^−1^ (positive control), RR-NPs, and nRR-NPs at 125 μg mL^−1^ or Complete Medium (negative control) for 36 minutes. The same experiment was repeated following exposition to Triton (*n* = 3) Values represent the mean ratio of fluorescent readings at Ex/Em 340/510 nm and 380/510 nm ± SEM (minimum of *n* = 3). Significance indicated by *** = *p* < 0.001 ** = *p* < 0.005 treatments compared with the untreated control.

An increase in intracellular calcium concentration ([Ca^2+^]^i^) can lead to the activation of sub-lethal cellular responses (such as cytokine production) *via* the stimulation of cell signalling cascades. In addition, an increase in [Ca^2+^]^i^ can cause lethal effects such as membrane damage, apoptosis or DNA damage.^31^ It was observed that [Ca^2+^]^i^ increased with time for all treatments (RR-NPs, nRR-NPs and UFCB) (Fig. 8) and that RR-NPs induced a greater response than nRR-NPs. Existing studies indicate that polymeric NPs can elicit an increase in [Ca^2+^]^i^ in a range of cell lines, however there are a lack of studies which have investigated this endpoint in hepatocytes. Polystyrene NPs can induce an increase in [Ca^2+^]^i^ in macrophages and neuroblasts.^32^ Furthermore, PLGA NPs increased intracellular calcium influx in both macrophage cells (RAW264.7) and human lung cells (BEAS-2B).^33^ Interestingly, primary rat hepatocytes have increased [Ca^2+^]^i^ when exposed to poly(amidoamine) (PAMAM) dendrimers with no impact on cell viability. However, this increase in [Ca^2+^]^i^ did not occur when cells were exposed to PEGylated PAMAM dendrimers.^34^

### Urea and albumin production

Quantification of urea and albumin production can be used as specific indicators of liver function *in vivo* and *in vitro*. Albumin is a protein made by the liver, while urea is formed when ammonia is detoxified in the liver.^35^

**Fig. 9:**
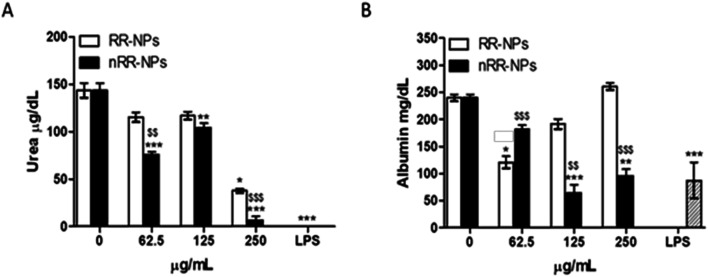
Urea and albumin production in C3A cells exposed to nRR-NPs and RR-NPs. Urea (A) and albumin (B) production following exposure of C3A hepatocytes for 24 h to RR-NPs and nRR-NPs at 62.5, 125 and 250 μg mL^−1^ or Complete Medium (0 μg mL^−1^ negative control). Data for urea are expressed as mean urea concentration (μg dL^−1^) ± SEM (*n* = 3). Data for albumin are expressed as mean albumin concentration (mg dL^−1^) ± SEM (*n* = 3). Significance indicated by *** = *p* < 0.001, ** = *p* < 0.005, * = *p* < 0.05 treatments compared with the untreated control. Significance indicated by $$$ = *p* < 0.001, $$ = *p* < 0.005 when comparing nRR-NPs to RR-NPs, at the same concentration.

Urea production was significantly reduced after exposure of cells to nRR-NPs, at all concentrations tested (Fig. 9). RR-NPs only decreased urea production at a concentration of 250 μg mL^−1^. Similarly, albumin production was significantly reduced by C3A cells exposed to nRR-NPs at 125 and 250 μg m^−1^, while RR-NPs only reduced albumin production at a concentration of 62.5 μg mL^−1^ (Fig. 9). These results indicate that both NPs may impair hepatocyte function and that nRR-NPs caused more damage to hepatocytes than RR-NPs. The impact of polymeric NPs on urea and albumin production by hepatocytes has not been investigated previously but we suggest it is more routinely assessed when assessing the hepatotoxicity of polymeric NPs *in vitro*.

## Conclusions

When assessing the safety of NPs it is critical to assess their hepatotoxicity to ensure their safe translation into the clinic. We used an *in vitro* model to assess the hepatotoxicity of polymeric NP delivery systems that can be targeted to cancer cells due to the incorporation of a redox sensitive element. It was demonstrated that NPs with and without a redox sensitive element exhibited low levels of cytotoxicity and that levels of cellular uptake were low. However, using a battery of tests we identified that both NPs activated sub-lethal responses in hepatocytes, with a greater toxic response observed for the nRR-NPs. More specifically, both NPs increased intracellular levels of calcium and ROS, and decreased hepatocyte function (urea and albumin production). Only nRR-NPs stimulated a genotoxic response, thus suggesting that the PEG loss of RR-NPs could modify the safety of the NPs due to different surface properties which impacts on their interactions with cells. Our findings can feed into the safe design of future polymeric NPs and provide insight into the mechanism of polymeric NP toxicity. More specifically, we demonstrated that polymeric NPs can increase ROS and calcium levels in cells which is likely to influence the cellular response that is activated. A greater understanding of the mechanism of polymeric NP toxicity will feed into the design of testing strategies which assess their safety.

## Author contributions

LGP, HJ and CC conceived and planned the experiments. LGP carried out the experiments. CC carried out sample preparation. LGP, HJ and CC contributed to the interpretation of the results. LGP led the writing the manuscript. LGP, HJ, CC, VS and CA provided critical feedback and helped shape the research, analysis, and manuscript.

## Conflicts of interest

The authors declare that there are no conflicts of interest.

## Supplementary Material
